# Editorial: Novel targeted drugs for indolent lymphoid malignancies

**DOI:** 10.3389/fonc.2023.1224528

**Published:** 2023-06-26

**Authors:** Tadeusz Robak, Bartosz Puła, Iwona Hus

**Affiliations:** ^1^ Department of Hematology, Medical University of Lodz, Lodz, Poland; ^2^ Department of General Hematology, Copernicus Memorial Hospital, Lodz, Poland; ^3^ Department of Hematology, Institute of Hematology and Transfusion Medicine, Warsaw, Poland; ^4^ Department of Hematology, National Medical Institute of the Ministry of Interior and Administration, Warsaw, Poland

**Keywords:** BRAF inhibitors, BTK inhibitors, CAR T, cellular therapies, hairy cell leukemia, follicular lymphoma, marginal zone lymphoma

Lymphoid malignancies, characterized by the uncontrolled proliferation of either mature B-lymphocytes or T-lymphocytes, can be classified as Hodgkin lymphoma or non-Hodgkin lymphoma (NHL). Slow-growing, mature NHLs can be further classified as indolent lymphoid malignancies based on their pathological, cytological and genetic features. In addition, the 5th Edition of the World Health Organization Classification and International Consensus Classification (ICC) recognize recognizes several mature B-cell neoplasms, including chronic lymphocytic leukemia/small lymphocytic lymphoma (CLL/SLL), follicular lymphoma (FL), marginal zone lymphoma (MZL), lymphoplasmacytic lymphoma, mantle cell lymphoma (MCL), and hairy cell leukemia (HCL), as well as various other rarer entities (1).

For many years, the standard of care in fit patients with indolent NHL was chemotherapy, and more recently, immunochemotherapy. However, targeted drugs have recently superseded immunochemotherapy in these patients, especially in a relapsed and refractory setting. Currently, most patients with relapsed or refractory indolent NHL receive Bruton’s tyrosine kinase (BTK) inhibitors, phosphatidylinositol 3–kinase inhibitors (PI3K) inhibitors, the BCL–2 inhibitor venetoclax, and CD20 monoclonal antibodies (Mab), used alone or in combination, such treatments are also becoming increasingly common in previously–untreated patients (2–4). The first BTK inhibitor approved for use by the US Food and Drug Administration (FDA) was ibrutinib, and since then, more selective next–generation iBTK inhibitors have been developed. Finally, CD19–directed chimeric antigen receptor T–cell (CAR T) therapies are now available for selected patients with relapsed and/or refractory (R/R) diffuse large B–cell lymphoma (DLBCL), primary mediastinal B–cell lymphoma (PMBCL), FL, and MCL (5). The most commonly–used targeted drugs for treating B–cell lymphoid malignancies are presented in **Figure 1**, together with their targets.

**Figure 1:**
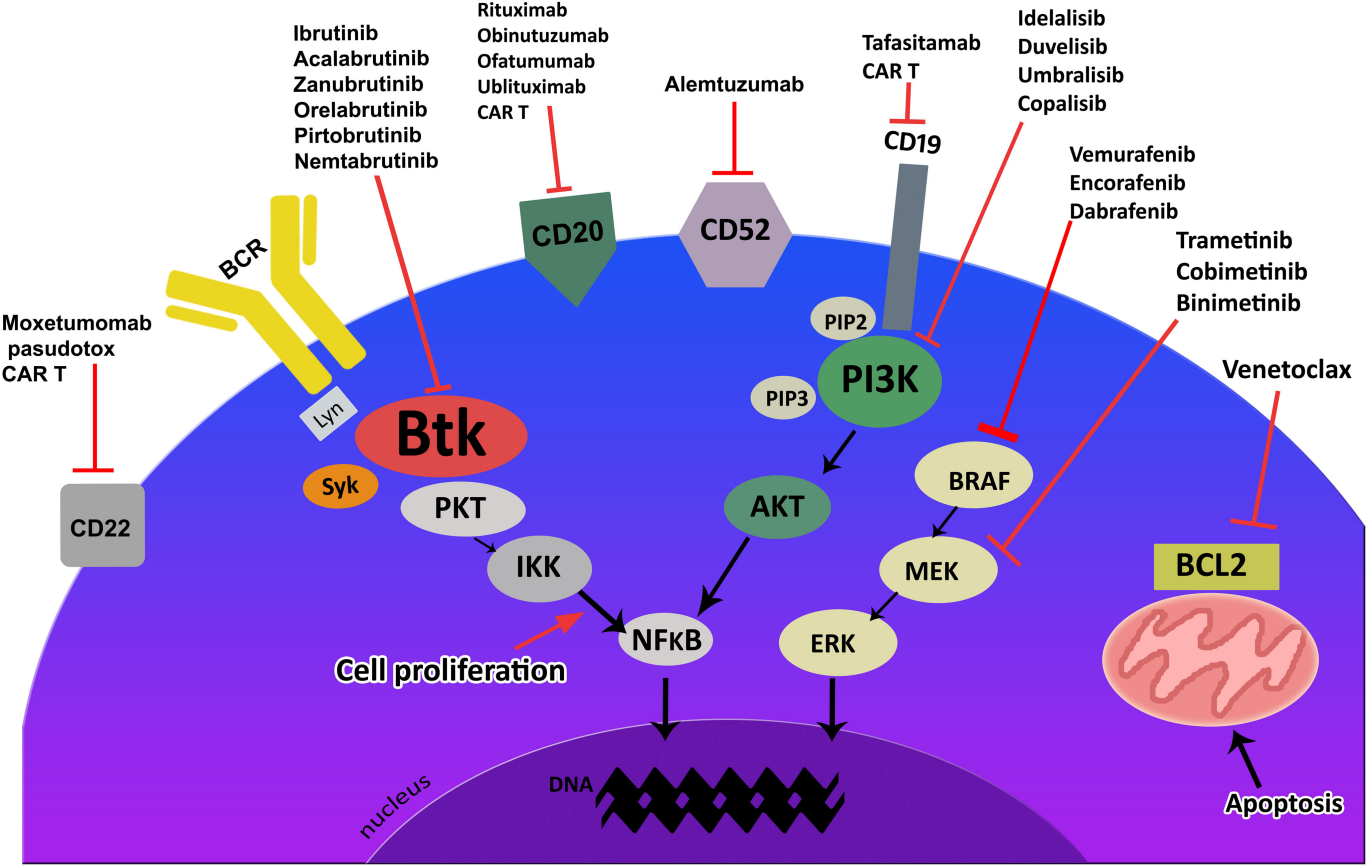
Targeted drugs and their targets for indolent B-cell lymphoid malignancies.

This Research Topic provides an excellent overview of the current status of targeted drugs in the treatment of indolent malignancies. The first review, by Deshpande and Munoz from the Mayo Clinic, presents the mechanisms of action and resistance of innovative therapeutic strategies with targeted and cellular therapies in B- and T-cell lymphoid malignancies. It discusses several Mabs, including the CD20 antibody rituximab, the immunotoxins brentuximab vedotin and polatuzumab vedotin, as well as nivolumab and pembrolizumab, which inhibit the programmed death receptor-1 (PD-1). It pays special attention to the clinical activity and resistance mechanisms of selected BTK inhibitors (ibrutinib), PI3K inhibitors (idelalisib, copanlisib) and BRAF inhibitors (vemurafenib). Finally, it discusses cellular therapy with currently-available CAR T-cell therapies and their FDA-approved clinical indications. Currently-approved CART cell therapies include six constructions (tisagenlecleucel, axicabtagene ciloleucel, brexucabtagene autoleucel, lisocabtagene maraleucel, idecabtagene vicleucel and citacabtagene autoleucel). CAR T-cell therapy has shown significant activity in patients with DLBCL, FL, MCL, B-cell acute lymphoblastic leukemia (ALL), and multiple myeloma. However, these therapies are associated with several toxicities, including cytokine release syndrome (CRS) and immune effector cell-associated neurotoxicity syndrome (ICANS). In addition, NHL patients treated with CAR T cells can develop resistance to therapy by altering CD19 frameshift mutations, leading to nonsense mutation-mediated CD19 decay or downregulation of CD20 expression in tumor cells. In addition, T-cell exhaustion can result in CAR T-cells demonstrating poor persistence after infusion and reduced clinical activity.

The article by Maitre et al. from the Hospitalier Universitaire Caen, France, discusses the results of recent clinical trials with targeted drugs in HCL. In this disease, recent discoveries in molecular biology have enabled the introduction of several targeted drugs for the treatment of patients refractory to the purine analogs, cladribine and pentostatin. In particular, recent clinical trials have confirmed the efficacy of BRAF inhibitors (vemurafenib, dabrafenib, encorafenib), MEK inhibitors (trametinib, cobametinib), BTK inhibitors, anti-CD20 Mab (rituximab) and CD22 immunotoxins (moxetumomab pasudotox). Among these new agents, the BRAF kinase inhibitors vemurafenib and dabrafenib are very active in patients with refractory and relapsed HCL and can be used in monotherapy or combination with CD20 Mabs or MEK inhibitors; however, deeper and longer responses can be achieved when vemurafenib is combined with rituximab. The BTK inhibitor ibrutinib is under investigation in patients with relapsed HCL and has demonstrated some activity. Moreover, other agents including cell cycle inhibitors (flavopiridol, palbocilib, abemacilib), epigenetic modifiers (5-azacitidine, decitabine, romidepsine, belinostat, panobinostat) and the EZH2 selective inhibitor tazemetostat are also in development in HCL.

Another original article by Song et al. from the Chinese Academy of Sciences, identifies a novel third-generation epidermal growth factor receptor (EGFR) inhibitor, ASK120067 (limertinib). Limertinib inhibits mutant EGFR by binding covalently to the kinase at cysteine 797 (Cys797) in the adenosine triphosphate (ATP)-binding domain. B. The authors found limertinib to inhibit BTK and interleukin-2-inducible T-cell kinase (ITK), and noted that this agent exhibited significant antitumour activities against B-cell lymphoma and T-cell leukemia *in vivo* and *in vitro* by targeting BTK and ITK in the preclinical models. In addition, limertinib irreversibly inhibits the kinases BTK/ITK more effectively than ibrutinib. These results suggest that limertinib merits further clinical trials in B-cell lymphoid neoplasms.

The next review article, by Wolska-Washer and Robak from the Medical University of Lodz, Poland, presents an overview of the clinical activity and safety of a second-generation irreversible BTK inhibitor, zanubrutinib, developed for lymphoid malignancies: a drug intended to reduce the adverse effects of ibrutinib. Zanubrutinib demonstrated more selective BTK inhibition than ibrutinib, with more complete and sustained BTK occupancy and improved oral absorption. Recent clinical trials have demonstrated excellent efficacy and good tolerability in patients with CLL, MCL, Waldenstrom macroglobulinemia and other lymphoid malignancies.

Finally, Rivero et al. from Hospital Clınic de Barcelona, Spain, summarize recent developments in the use of targeted drugs in the treatment of FL and MCL. The authors present clinical data on approved and emerging novel targeted therapies, including PI3K inhibitors, BTK inhibitors, EZH2 inhibitors, Mabs and immunotoxins. They also discuss the activity of lenalidomide combinations, bispecific T-cell engagers and CAR T therapy.

In conclusion, this Research Topic provides an excellent platform for a better understanding of novel targeted therapies in indolent lymphoid malignancies. We would like to thank all of the authors included herein, for their high-quality articles, as well as the reviewers for their professional article evaluation, and the editorial staff for their support.

## Author contributions

All authors listed have made a substantial, direct, and intellectual contribution to the work and approved it for publication.
